# Mutation in xyloglucan 6-xylosytransferase results in abnormal root hair development in *Oryza sativa*

**DOI:** 10.1093/jxb/eru189

**Published:** 2014-05-15

**Authors:** Chuang Wang, Shuai Li, Sophia Ng, Baocai Zhang, Yihua Zhou, James Whelan, Ping Wu, Huixia Shou

**Affiliations:** ^1^State Key Laboratory of Plant Physiology and Biochemistry, College of Life Sciences, Zhejiang University, 388 Yuhangtang Road, Hangzhou, 310058, P. R. China; ^2^University of Western Australia-Zhejiang University Joint Research Laboratory in Genomics and Nutriomics, College of Life Sciences, Zhejiang University, 388 Yuhangtang Road, Hangzhou, 310058, P. R. China; ^3^ARC Centre of Excellence in Plant Energy Biology, The University of Western Australia, 35 Stirling Highway, Crawley, 6009 Western Australia, Australia; ^4^State Key Laboratory of Plant Genomics, Institute of Genetics and Developmental Biology, Chinese Academy of Sciences, Beijing 100101, China; ^5^Department of Botany, School of Life Science, Australian Research Council Centre of Excellence in Plant Energy Biology, La Trobe University, Bundara, Victoria 3086, Australia

**Keywords:** Rice, root hair, type I cell wall, type II cell wall, xyloglucan, xylosyltransferase.

## Abstract

Root hairs are important for nutrient uptake, anchorage, and plant–microbe interactions. From a population of rice (*Oryza sativa*) mutagenized by ethyl methanesulfonate (EMS), a *short root hair2* (*srh2*) mutant was identified. In hydroponic culture, *srh2* seedlings were significantly reduced in root hair length. Bubble-like extrusions and irregular epidermal cells were observed at the tips of *srh2* root hairs when grown under acidic conditions, suggesting the possible reduction of the tensile strength of the cell wall in this mutant. Map-based cloning identified a mutation in the gene encoding xyloglucan (XyG) 6-xylosyltransferase (*OsXXT1*). OsXXT1 displays more than 70% amino acid sequence identity with the previously characterized *Arabidopsis thaliana* XYG XYLOSYL TRANSFERASE 1 (AtXXT1) and XYG XYLOSYL TRANSFERASE 2 (AtXXT2), which catalyse the transfer of xylose onto β-1,4-glucan chains. Furthermore, expression of the full-length coding sequence of *OsXXT1* could complement the root hair defect, and slow growth and XyG synthesis in the *Arabidopsis xxt1 xxt2* double mutant. Transgenic plants expressing the β-glucuronidase (GUS) reporter under the control of the *OsXXT1* promoter displayed GUS expression in multiple tissues, most prominently in root epidermal cells. These results demonstrate the importance of *OsXXT1* in maintaining cell wall structure and tensile strength in rice, a typical grass species that contains relatively low XyG content in cell walls.

## Summary

*OsXXT1* showed xyloglucan 6-xylosyltransferase activity and is involved in maintaining cell wall structure and tensile strength in rice. Mutation of *OsXXT1* results in abnormal root hair development.

## Introduction

Plant cell walls are composed of cellulose, hemicellulose, and pectin, in addition to a number of inorganic compounds. Based on the chemical structures, wall architecture, and cell wall biosynthetic processes, primary cell walls of flowering plants are divided into two classes, type I and type II (Carpita and Gibeaut., 1993; Carpita, 1996; Carpita and McCann, 2000). Type I cell walls are typically found in dicots and non-commelinoid monocots. They are characterized by a cellulose–xyloglucan (XyG) network with high pectin and structural proteins content. Type II cell walls are found only in the commelinoid monocots (e.g. grasses, rushes, and gingers) and are composed of cellulose fibres encased in glucuronoarabinoxylans (GAX), high levels of hydroxycinnamates, and very low levels of pectin and structural proteins. Additionally, cell walls of grasses and some related families contain significant amounts of mixed linkage glucans (Carpita, 1996; Carpita and McCann, 2000; Vogel, 2008).

**Fig. 1.**
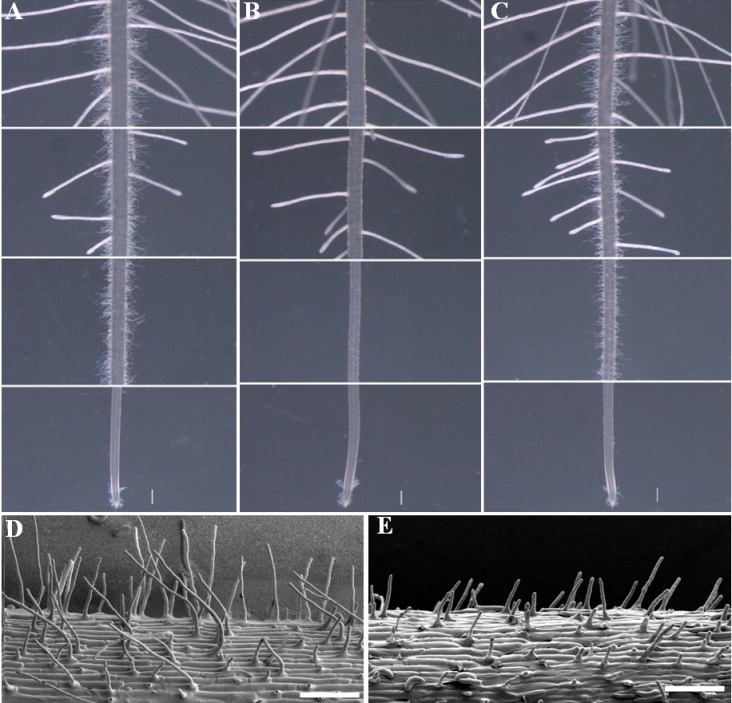
Phenotype of root hairs from wild type (WT, cv Kasalath), *srh2*, and *srh2* complemented by *Ubiquitin-1*_*promoter*_::*OsXXT1*. (A and D) root hairs from the WT; (B and E) root hairs from *srh2*; (C) root hairs from transgenic seedling of *srh2* overexpressing *OsXXT1*. Seedlings of A, B, and C were grown under the same pots with nutrient solution for seven days. Bar=1cm. Seedlings of D and E were grown for three days on Murashige and Skoog medium (pH 5.5) and examined under an electron microscope examination. Bar=200 μm.

XyG is the most abundant hemicellulosic polysaccharide in type I cell walls, which comprises ~20–25% dry mass of cell walls (Obel *et al.*, 2007; Vogel, 2008). XyG consists of a β-1,4-glucan backbone with α-d-xylose substitution on the oxygen-6 position in a regular pattern. There are two general types of XyG: the poly-XXXG and poly-XXGG; approximately 75% and 50% of their backbone residues are branched, respectively (Vincken *et al.*, 1997). These xylosyl residues can be further substituted at the oxygen-2 position with either a single β-D-galactose or an α-L-fucose-(1,2)-β-d-galactose dimer, depending on the plant species (Obel *et al.*, 2007). The sub-structure of XyG has been investigated in detail using β-1,4-endoglucanase to digest the non-substituted glucose units in the polymer (Hoffman *et al.*, 2005). Hydrolysed *Arabidopsis* XyG released XXXG, XLXG, XXFG, XLLG, and XLFG subunits (see Figure 1 and 2 in Obel *et al.* (2007) for a description of XyG nomenclature). In contrast to the observations made in *Arabidopsis,* the hemicelluloses of primary cell walls in graminaceous monocots are mainly comprised of xylans and mixed linkage glucans. Only ~1–5% of XyG is found in cereals or grasses with a low degree of substitution (Carpita, 1996; Hoffman *et al.*, 2005; Sims *et al.*, 2000).

**Fig. 2.**
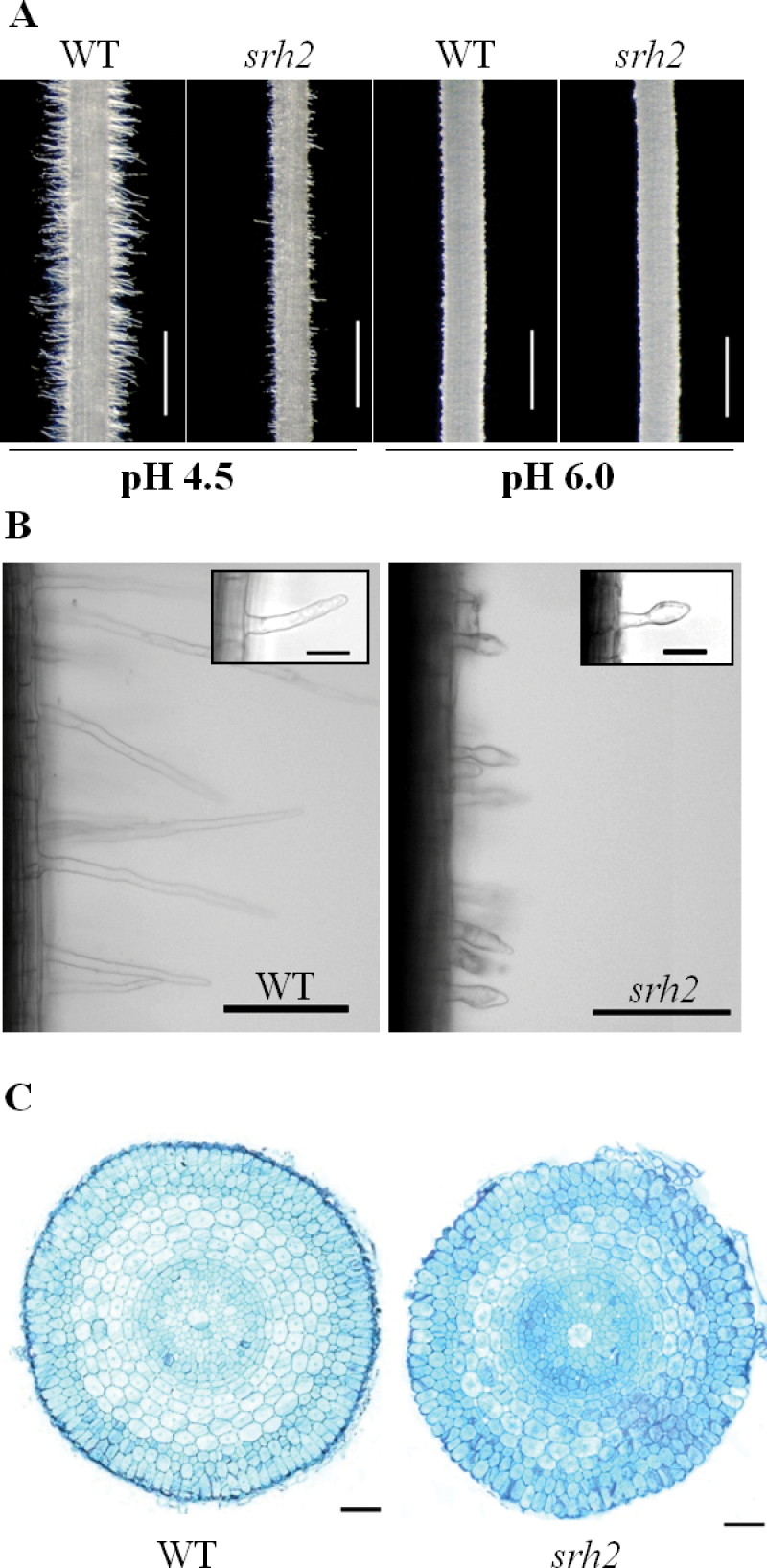
Root hair morphology of rice seedlings grown in different media with various pH. (A) Root hairs of three-day-old seedlings of wild-type (WT) and *srh2* grow on Murashige and Skoog medium at pH 4.5 and 6.0. Bar=1mm. (B) The root hairs of the WT and *srh2* examined under a microscope (pH 4.5). Bar=100 μm. (C) Cross section of root tip from WT and *srh2* seedlings grown at pH 4.5. The section was strained with toluidine blue (TBO). Bar=20 μm. (This figure is available in colour at *JXB* online.)

Although the cell wall composition differs between type I and type II cell walls, many cell wall-related genes are conserved between species with both types, presumably to maintain the basic structure of cell walls (Penning *et al.*, 2009; Yokoyama and Nishitani, 2004). The β-glucan backbone of XyG is synthesized by a cellulose synthase-like C family protein, named CSLC4 in *Nasturtium* and *Arabidopsis* (Cocuron *et al.*, 2007). AtXXT1, AtXXT2, and AtXXT5 are involved in linking xylose to the β-glucan backbone at different positions (Cavalier and Keegstra, 2006; Cavalier *et al.*, 2008; Faik *et al.*, 2002; Vuttipongchaikij *et al.*, 2012; Zabotina *et al.*, 2008; Zabotina *et al.*, 2012). The galactosyltransferase MUR3 and fucosyltransferase MUR2 further catalyse the UDP–galactose or UDP–fucose to the side chain of a XyG oligosaccharide block (Madson *et al.*, 2003; Vanzin *et al.*, 2002). Plants with mutations affecting XyG structure or content exhibited collapsed trichome papillae or abnormal root hairs (Cavalier *et al.*, 2008; Madson *et al.*, 2003; Pena *et al.*, 2012; Vanzin *et al.*, 2002; Zabotina *et al.*, 2008), and Arabidopsis XyG mutants had reduced tensile strength in primary cell walls (Cavalier *et al.*, 2008; Park and Cosgrove, 2012a; Pena *et al.*, 2004).

Grass cell walls are a major source of dietary fibre for animals and a significant source of renewable energy (Ragauskas *et al.*, 2006). Cell walls in grass species contain significant amounts of GAX and mixed linkage glucans, but relatively low amounts of XyG. It is commonly accepted that XyG is a less important component in type II cell walls compared with type I cell walls (Vogel, 2008). The function and genetic controls of synthesis of XyG is unknown in type II cell walls.

In this study, we isolated and characterized the rice *short root hair2* (*srh2*) mutant and provide evidence for the importance of XyG in cell wall structure and root hair tip growth in a grass species.

## Materials and methods

### Plant materials and growth conditions

The rice *srh2* mutant was identified in an EMS-mutagenized population from the rice cultivar Kasalath. For all experiments, the *srh2* mutant and wild-type seeds were germinated in distilled water for two days. The seedlings were then grown in a hydroponic solution (Yoshida *et al.*, 1976). Seedlings were grown in a growth chamber at 30 °C/22 °C day/night temperatures with a 12h light/12h dark regime (450 μmol photons m^–2^s^–1^). For the pH treatment experiment, seeds were surface sterilized with 95% (*v/v*) ethanol for 2min and 15 % (*v/v*) bleach for 20min. After rinsing in distilled water, seeds were germinated and grown in test tubes (15 cm×3cm) containing Murashige and Skoog medium, 0.059% (*w/v*) 2-(*N*-morpholino) ethanesulfonic acid (MES), 1% (*w/v*) sucrose and 0.3% (*w/v*) phytagel (Sigma, US). The basic medium contained 2.0mM NH_4_NO_3_, 1.9mM KNO_3_, 0.3mM CaCl_2_·2H_2_O, 0.15mM MgSO_4_·7H_2_O, 5 μM KI, 25 μM H_3_BO_3_, 0.1mM MnSO_4_·H_2_O, 0.3mM ZnSO_4_·7H_2_O, 1 μM Na_2_MO_4_·2H_2_O, 0.1 μMCuSO_4_·5H_2_O, 0.1 μM CoCl_2_·6H_2_O, 0.1mM FeSO_4_·7H_2_O, and 0.1mM Na_2_EDTA·2H_2_O.

### Map-based cloning and genetic complementation

An F_2_ mapping population was generated from crosses between homozygous *srh2* mutant plants and the Japonica cultivar Nipponbare. The *SRH2* gene was mapped to chromosome 3 between simple sequence repeat (SSR) markers RM232 and RM3280 using 1800 F_2_ mutant plants. SSR markers were obtained from NCBI database (http://www.ncbi.nlm.nih.gov/unists). The mutation was further mapped to a 36-kb region between STS274-04 and STS274-04-06 using nine newly developed SSR markers. Based on the phenotype of *srh2*, the *OsXXT1* gene was selected as a candidate gene. A derived cleaved amplified polymorphic sequences (dCAPS) marker was developed using the dCAPS finder 2.0 program (http://helix.wustl.edu/dcaps/dcaps.html) to further confirm the mapping result. The genes were amplified by PCR from genomic DNA isolated from *srh2* and wild-type plants and sequenced to identify the mutation in the genomic sequence.

For complementation, the full-length open reading frame of *OsXXT1* was amplified by reverse transcription PCR and inserted into the modified binary vector pTF101-ubi (Zheng *et al.*, 2010) between the maize *Ubiquitin-1* promoter and a nopaline synthase terminator. The resulting transformation plasmid, pXXT1-Oe, was used for the *Agrobacterium*-mediated rice transformation of *srh2* mutant as described (Nishimura *et al.*, 2006).

For the complementation of *Arabidopsis xxt1 xxt2* double mutant, the full length coding sequence of OsXXT1 was PCR amplified and cloned into the binary vector (pH7WG2D). The expression of OsXXT1 was driven by the constitutive 35S CaMV promoter. This binary vector was then transformed into *Agrobacterium tumefaciens* GV3130 strain. Floral dipping was performed with an inoculum medium containing 10% (*w/v*) sucrose and 0.05% (*v/v*) Silwet-77 (Clough, 1998). T1 transformants were screened on hygromycin (50mg l^–1^) (Harrison, 2006). Successful transformants, were age matched and Columbia-0 and *Arabidopsis xxt1 xxt2* double mutant plants were used for imaging using a Nikon Eclipse 80i Microscope (Nikon, Japan).

### Microscopic analysis

The root hairs were examined by a Leica MZ95 stereomicroscope with a colour CCD camera (Leica Instrument, Nusslosh, Germany). For cryo-scanning electron microscopy seeds were plated on Murashige and Skoog medium and grown for three days (pH 5.7). Root samples were placed on moist nitrocellulose paper mounted on a stub and immersed in liquid nitrogen slush, then transported under vacuum to a cryo preparation chamber. Ice was sublimed at –90 °C and the specimens were sputter-coated with gold and observed using a Hitachi S-3000N scanning electron microscope (Hitachi, Naka, Japan) with a Gatan Alto 2100 cryo preparation system (Gatan UK, Abingdon, UK).

For light microscopic analysis, root tips were fixed overnight in 2.5% (*v/v*) glutaraldehyde with 0.1M sodium phosphate buffer (pH 7.2) and washed three times for 30min in the same buffer. Root samples were then re-fixed for 4h in 1% (*v/v*) OsO_4_ with 0.1M sodium phosphate buffer (pH 7.2) and washed for 30min in the same buffer. The samples were dehydrated in a gradient ethanol and embedded in Spurr resin. Semi-thin sections (2 μm) were made using glass knives on a Power Tome XL (RMC-Boeckeler Instruments, Arizona, USA) microtome and stained in 0.1% (*w/v*) methylene blue for 3min at 70 °C. The samples were rinsed with distilled water and visualized with a Zeiss Axiovert 200 microscope (Zeiss, Jena, Germany).

For GUS staining, tissues samples were fixed overnight in FAA (constituted of 5% (*v/v*) formalin, 5% (*v/v*) acetic acid, 75% (*v/v*) alcohol) and washed twice for 30min in 70% (*v/v*) ethanol. The tissues were dehydrated in gradient acetone and embedded in Spurr resin. The sections and visualizations were carried out as described above.

### Sequence alignments and phylogenic analysis

The putative XXT sequences for alignment were extracted from NCBI (http://www.ncbi.nlm.nih.gov/). Multiple sequence alignment of XXT proteins was conducted using the ClustalX 1.83 program (Thompson *et al.*, 1997) with default multiple alignment parameters and viewed by GeneDoc 3.2. A phylogenic tree of the gene family was constructed using the Neighbor–Joining method by MEGA5.

### Matrix-assisted laser desorption/ionizationd time-of-flight (MALDI-TOF) mass spectrometry analysis of xyloglucan oligosaccharides

Cell wall was extracted from leaves that were ground into powder in liquid nitrogen. The homogenate was washed three times with hot 70% (*v/v*) ethanol and extracted with a mixture of chloroform and methanol (1:1). The pellet was suspended in acetone and air-dried overnight. The alcohol-insoluble residues (AIRs) were de-starched with α-amylase (Bacillus sp). The XyG enriched KOH-soluble fraction was prepared by neutralizing 50mg of de-starched AIRs in 4M KOH solution, samples were then dialysed and finally lyophilized. Then, 0.5mg of AIRs or KOH fraction was incubated in 100ml of 50mM ammonium formate, pH 5.0, with one unit of xyloglucanase (EXEGP; Megazyme) for 18h at 37 °C. The supernatants were recovered, and 1ml of aqueous sample plus 10ng xylopentaose was spotted with an equal volume of matrix solution (10mg ml^–1^ 2,5-dihydroxbenzoic acid). After being dried on the MALDI target plate, spectra were analysed on a Bruker Autoflex MALDI-TOF mass spectrometry instrument (Bruker) in the positive reflection mode with an acceleration voltage of 20kV. The relative height of each generated oligosaccharide ion peak was counted to determine their relative abundance as described previously (Zhang *et al.*, 2012).

### Generation of OsXXT1::GUS transgenic lines

The 1.8-kb region upstream of the start codon of *OsXXT1* gene was amplified from the genomic DNA of Kasalath using primers shown in Table S1. The PCR product was then cloned into the pBIGUS-plus vector, in which the original *GUS* gene in binary vector pBI101.3 was replaced by a GUS-Plus sequence from pCAMBIA1305.1. The resultant vector was introduced into Nipponbare rice using *Agrobacterium*-mediated rice transformation as described (Nishimura *et al.*, 2006).

### GUS histochemical analysis

Histochemical GUS staining was performed as described in Jefferson *et al.* (1987). Plant tissues from GUS transgenic lines were immediately submerged in GUS staining solution after harvest and placed under a vacuum for 10min. The samples were incubated overnight in darkness at 37 °C. Chlorophyll was removed by submerging the stained tissues in 70% (*v/v*) ethanol. Plant material was placed on glass slides using 20% chloral (*w/v*) in 25% glycerol (*v/v*) for 10min. GUS staining was visualized using a Leica MZ95 stereomicroscope with a colour CCD camera (Leica Instrument, Nusslosh, Germany). The GUS staining solution contained 100mM sodium phosphate buffer (pH 7.0), 10mM Na_2_EDTA, 1mM K_3_[Fe(CN_6_)], 1mM K_4_[Fe(CN_6_)], 0.5% (*v/v*) TritonX-100, 20% (*v/v*) methanol, and 0.5mg ml^–1^ 5-bromo-4-chloro-3-indolyl-β-d-glucuronic acid (X-gluc).

### Expression analysis

Publically available Affymetrix *Arabidopsis* and rice microarray CEL files were downloaded from the Gene Expression Omnibus within the National Centre for Biotechnology Information database or from the MIAME ArrayExpress database (http://www.ebi.ac.uk/arrayexpress/). The CEL files were imported and quantile normalized together using Partek Genomics Suite version 6.5 (St. Louis, Missouri, USA) as carried out in previous studies (Narsai *et al.*, 2011). The accession numbers for the *Arabidopsis* studies were GSE30223 and E-AFMX-9, and for rice several were combined, including E-MEXP-1766, E-MEXP-2267, GSE6908, GSE11966, GSE7951, and GSE6893.

## Results

### Isolation of the *srh2* mutant

Seeds from the M_2_ generation of an EMS-mutagenized population of Indica cultivar Kasalath, were germinated and grown in nutrient solution to screen for mutants with abnormal root hair phenotype. Seven days after germination, a mutant with significantly reduced length in root hairs was identified (Fig. 1A, B). No obvious difference was observed in leaf or root growth between wild type and mutant (Fig. S1). The mutant was designated as *srh2*. Scanning electron microscopy of the wild-type and *srh2* root surface showed that the length of the root hairs of *srh2* were shorter than those of wild type. However, no significant difference was found in root hair density or distribution pattern between *srh2* and wild type (Fig. 1D, E).

It is reported that extracellular pH can regulate root hair growth by modification to cell wall rigidity (Monshausen *et al.*, 2007). To investigate whether the root hair growth of *srh2* is affected by extracellular pH, wild-type and mutant seeds were germinated on Murashige and Skoog medium under pH 4.5 and 6.0. The medium pH was stabilised by MES. At pH 6.0, root hair growth in both wild type and the *srh2* mutant was significantly inhibited (Fig. 2A). The acidic conditions (pH 4.5) induced root hair growth in wild-type seedlings, but not in the *srh2* mutant (Fig. 2A). Bubble-like extrusions were observed at the tip of the hairs in the *srh2* mutants grown under acidic conditions (Fig. 2B). Transverse section of the wild-type and mutant roots showed that the shape of epidermal cells in the *srh2* root meristem zone was irregular (Fig. 2C).

### Gene cloning of the *srh2* mutant

**Fig. 3.**
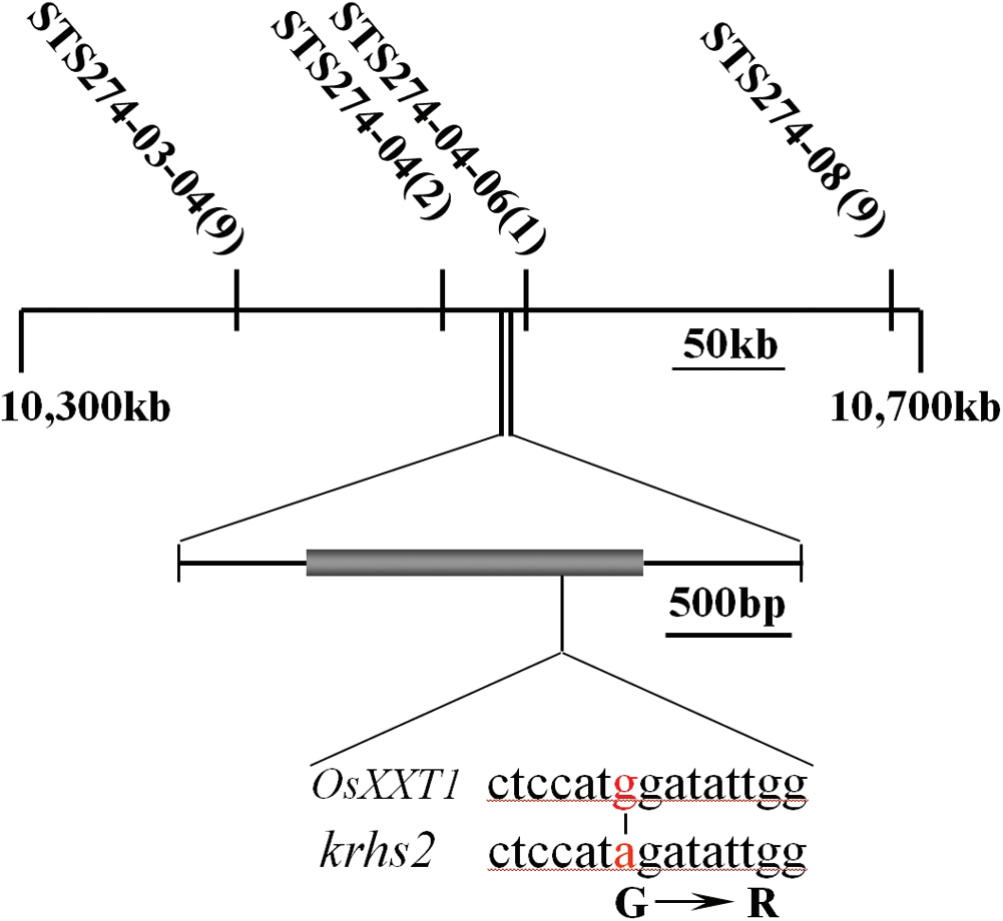
Molecular identification of *srh2* by positional cloning. Physical map of the chromosomal region encompassing the *srh2* gene was defined by high-resolution mapping. *srh2* was mapped between the simple sequence repeat (SSR) markers STS274-04 and STS274-04-06 with the number of recombinants given in parentheses. (This figure is available in colour at *JXB* online.)

Genetic analysis showed that a single recessive gene was responsible for the mutant phenotype. In 1800 mutant seedlings from the F2 population derived from a cross between the mutant (from the Indica cultivar Kasalath) and the Japonica cultivar Nipponbare, the roots hairs of 1362 seedlings grew normally, whereas 438 seedlings showed short root hairs. Using this population, the mutation was mapped to a 36-kb region between SSR markers STS274-04 and STS274-04-06 on chromosome 3 (Fig. 3). This region contains seven open reading frames, including a putative *OsXXT1* gene (LOC_Os03g18820). Altered XyG structure or content of cell walls in *Arabidopsis* caused collapsed trichome papillae or a short root hair phenotype (Cavalier *et al.*, 2008; Madson *et al.*, 2003; Vanzin *et al.*, 2002; Zabotina *et al.*, 2012). Therefore, the phenotype of *srh2* suggested that the *OsXXT1* gene is a tentative candidate gene for the mutation. To confirm this, the full-length cDNA sequence of the *OsXXT1* gene was amplified from both the wild-type and *srh2* mutant genomic DNA. Comparison of these two sequences revealed the presence of a single point mutation (G to A) at the nucleotide position 1009bp from the start codon of *OsXXT1* (Fig. 3). The nucleotide substitution of the *srh2* mutant sequence resulted in an amino acid change of a glycine (G) into an arginine (R) (Fig. 3). To further verify the positional cloning of the mutant, a dCAPS marker was developed using the *Nco*I restriction endonuclease (Fig. S2). The enzyme cuts the PCR product of the wild-type *OsXXT1* gene into four fragments: 456bp, 73bp, 244bp, and 1210bp. In contrast, the PCR product amplified from the *srh2* mutant produced only three fragments when digested with *Nco*I: 529bp, 244bp, and 1210bp (Fig. S2).

A genetic complementation test was carried out to confirm that the point mutation in *OsXXT1* was responsible for the mutant phenotype. The full-length open reading frame of the wild-type *OsXXT1* gene was inserted into the binary vector pTF101.1 under the control of a maize *Ubiquitin-1* promoter. The resulting construct was used to introduce a full-length *OsXXT1* gene into the *srh2* mutant genome via *Agrobacterium*-mediated transformation. Four positive transgenic lines were identified. The root hair from the T_2_ transgenic seedlings displayed normal root hair growth (Fig. 1C), indicating overexpression of the *OsXXT1* gene could complement the mutant phenotype completely.

### Protein structure and phylogenetic analysis of OsXXT1

**Fig. 4.**
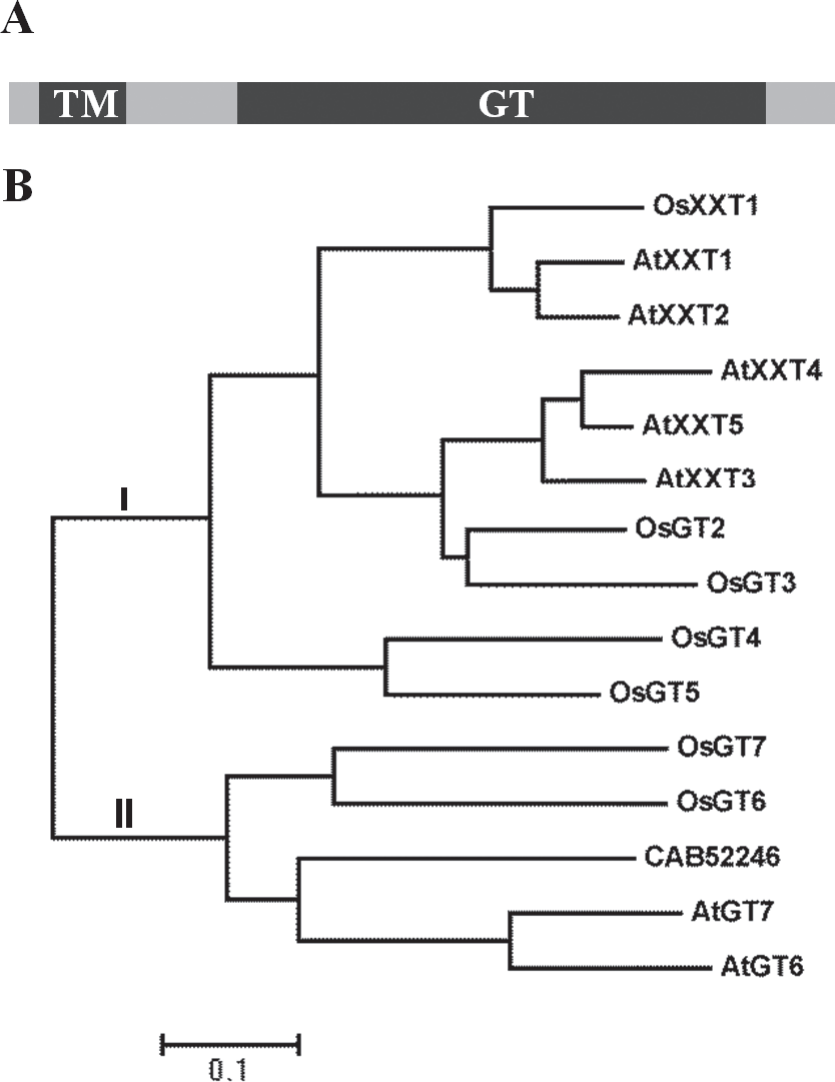
Schematic diagram of protein domain structure and phylogenetic analysis of OsXXT1. (A) Predicted schematic of OsXXT1 protein domain structure. TM, predicted transmembrane domain; GT, predicted glycosyltransferase domain. (B) Neighbor–joining phylogenic tree of putative xylosyltransferases in *Arabidopsis* and rice using MEGA5 program. CAB52246 is accession number of fenugreek α-(1,6) galactosyltransferase. The gene locus of rice GT34 genes are: *OsXXT1*, LOC_Os03g18820; *OsGT2*, LOC_Os02g32750; *OsGT3*, LOC_Os12g05380; *OsGT4*, LOC_Os03g19310; *OsGT5*, LOC_Os03g19330; OsGT6, LOC_Os11g34390; *OsGT7*, LOC_Os02g49140.

The *OsXXT1* gene encodes a predicted protein of 448 amino acids that has been classified as a member of glycosyltransferase family 34 (GT34) in CAZy (http://www.cazy.org). The OsXXT1 protein is predicted to have a transmembrane domain near the N-terminus and a glycosyltransferase domain near the C-terminus (Fig. 4A). The base substitution of the *srh2* mutant sequence resulted in an amino acid change of a glycine to an arginine in the highly conserved glycosyltransferase domain (Fig. S3).

Phylogenetic analysis of 14 putative xylosyltransferases in *Arabidopsis* and rice revealed that these genes could be divided into two classes (Fig. 4B). Five genes from rice, and five genes from *Arabidopsis*—AtXXT1 to AtXXT5, which have demonstrated XXT activity—are grouped into class I. Class II contains two *Arabidopsis* genes (*AtGT6* and *AtGT7*) and two rice genes (*OsGT6* and *OsGT7*), which are clustered with the fenugreek α-(1,6) galactosyltransferase (*CAB52246*). Although there are 8, 10, and 18 RNA variants encoding GT34 protein members in *Arabidopsis*, rice, and maize (Penning *et al.*, 2009), respectively, only class I proteins contained both the transmembrane and glycosyltransferase domains in *Arabidopsis* and rice (Fig. S3, and Fig. 4B).

### Functional verification of OsXXT1 by complementation of the *Arabidopsis xxt1 xxt2* double mutant

**Fig. 5.**
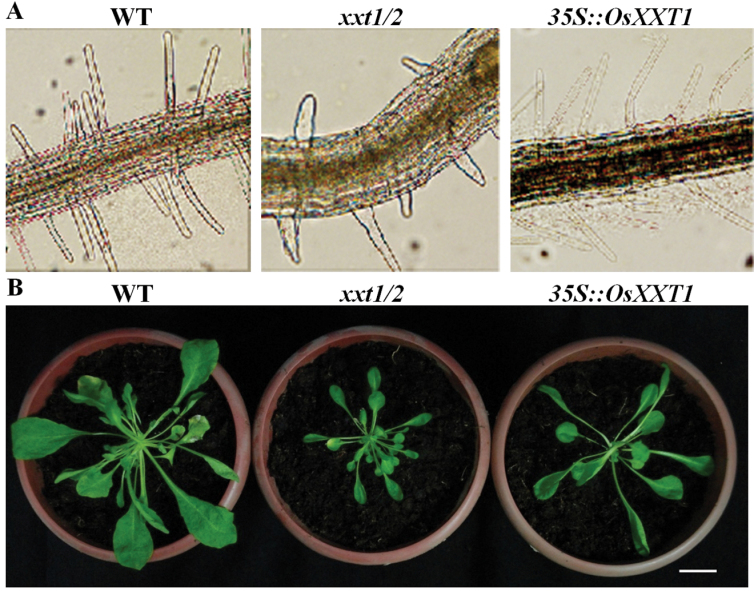
Complementation of root hair and slow growth phenotype of *Arabidopsis xxt1 xxt2* double mutant with *OsXXT1* in ten-day-old seedlings. Representative examples of root hair and growth are shown. (A) Roots hairs of wild type (WT, Columbia-0), *Arabidopsis xxt1 xxt2* double mutant, and complemented *Arabidopsis xxt1 xxt2* double mutant with *35S::OsXXT1* (*35S::OsXXT1*). Bars=100 μm. (B) Seedlings of wild type (WT, Columbia-0), *Arabidopsis xxt1xxt2* double mutant and *35S::OsXXT1*. Bars=2cm. (This figure is available in colour at *JXB* online.)

**Fig. 6.**
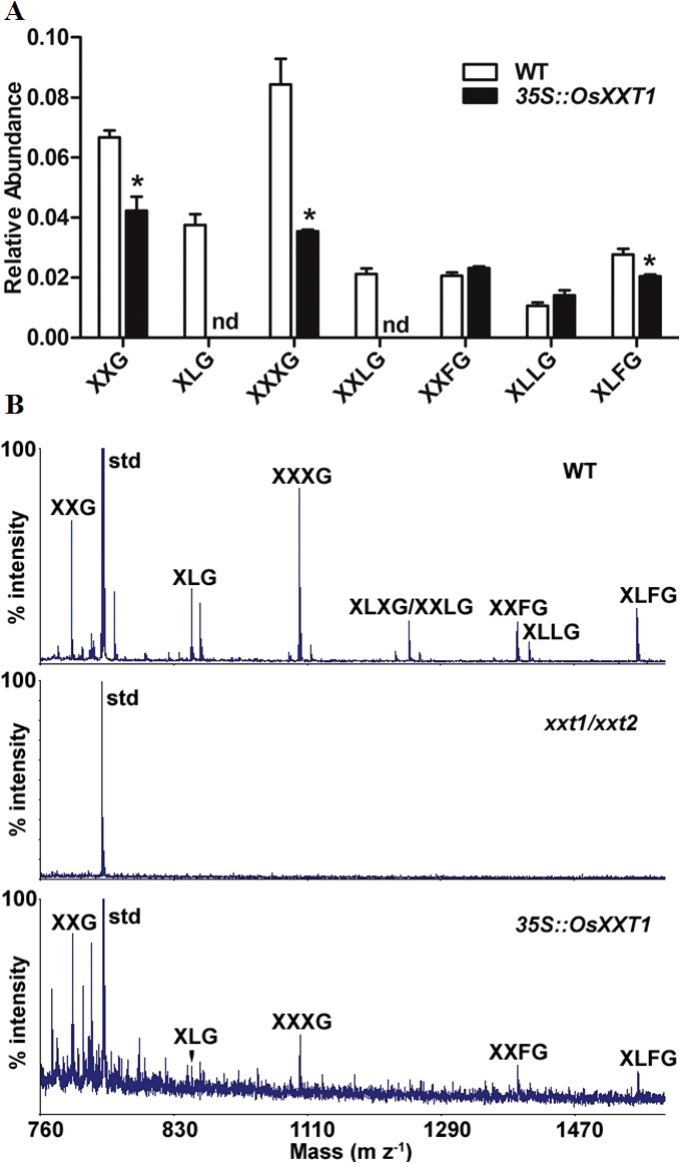
MALDI-TOF mass spectrometry analysis of the relative abundance of xyloglucan oligosaccharides released by xyloglucan-specific endoglucanase. (XEG). (A) Relative proportions of xyloglucan subunits generated from cell wall preparations of wild-type (WT, white bar) and *35S::OsXXT1 Arabidopsis* (black bar) leaves digested with XEG. Results are expressed as the percentile of the areas of the corresponding peaks for each subunit on high-performance anion exchange chromatography (HPAEC). *Arabidopsis* were grown in growth chamber for two months and leaves of each of the plants were sampled as a biological replicate. All analyses were performed on three plant preparations. Significant differences are indicated with an asterisk. nd, not detected. (B) MALDI-TOF mass spectrometry of XEG-generated xyloglucan fragments from the hemicellulosic fractions of wild-type (WT, Columbia-0), *Arabidopsis xxt1 xxt2* double mutant, and complemented *Arabidopsis xxt1 xxt2* double mutant with *35S::OsXXT1* (*35S::OsXXT1*). Xyloglucan subunit is described from the nomenclature introduced by Fry *et al.* (1993). WT (top), *Arabidopsis xxt1 xxt2* (middle) and *35S::OsXXT1* (bottom). Xylopentaose was used as a standard control (std).

The amino acid sequence of OsXXT1 is 70% and 72% identical to that of AtXXT1 and AtXXT2 in *Arabidopsis*, respectively. This suggests that *OsXXT1* functions as an *XXT* gene in rice, as *AtXXT1* and *AtXXT2* function in *Arabidopsis*. To test if OsXXT1 possesses xyloglucan 6-xylosytransferase activity, a functional complementation test of the *Arabidopsis xxt1 xxt2* double mutant, which displays a root hair development phenotype (Cavalier *et al.*, 2008), was carried out. Expression of *OsXXT1* under the 35S CaMV promoter was measured by reverse-transcription PCR (RT-PCR) in complemented transgenic lines (*35S::OsXXT1*). Two transgenic lines showed a high expression level of *OsXXT1* (Fig. S4), and in these two lines the short root hair growth was restored (Fig. 5A). Furthermore, the expression of *OsXXT1* also partially complemented the slow growth phenotype of the *Arabidopsis xxt1 xxt2* double mutant (Fig. 5B). Thus, OsXXT1 may have xyloglucan 6-xylosytransferase similar to AtXXT1 and AtXXT2. To measure the effect of *OsXXT1* on XyG synthesis, cell walls from the leaves of wild type, *xxt1 xxt2* double mutant and *35S::OsXXT1*-complemented *Arabidopsis* plants were digested with a XyG-specific endoglucanase (XEG) followed by extraction with 4M KOH. Xyloglucan oligosaccharides released after XEG digestion were analysed by matrix-assisted laser desorption/ionization time-of-flight (MALDI-TOF) mass spectrometry (Fig. 6B). As previously reported, we could not detect any signature of XyG fragment in the *Arabidopsis xxt1 xxt2* mutant (Fig. 6B). In the *35S::OsXXT1*-complemented *Arabidopsis* lines, we detected several XyG oligosaccharides, including normal wild-type levels of XXFG, XLLG, and XLFG, and relatively lower level of XXG, XLG, XXXG, and XXLG compared with wild-type plants (Fig. 6A, B).

### Expression pattern of OsXXT1

**Fig. 7.**
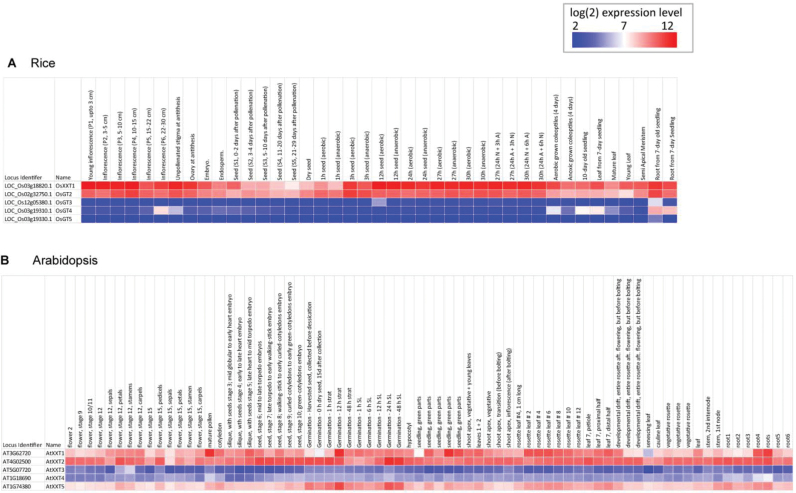
Tissue-specific expression of rice XXT genes from rice (A) and *Arabidopsis* (B). The averaged normalized publically available expression data across development in both species is shown. Data are reported as log_2_ average intensities on a heatmap, whereby low expression is indicated by blue shading and higher expression indicated by red shading.

Expression patterns of rice *XXT* subfamily genes were determined by examining expression across development using publically available microarray datasets. The expression data are colour coded according to the RNA expression level (Fig. 7). *OsXXT1* and *OsGT2* have similar expression patterns and are expressed across a variety of tissues. In contrast, other members of this family (*OsGT3* to *OSGT5*) display a more restricted expression pattern, suggesting that *OsXXT1* and *OsGT2* are the predominantly expressed genes (Fig. 7A). A comparison to the expression in *Arabidopsis* displays a similar pattern, with *AtXXT1* and *AtXXT2* being predominantly expressed, followed by *AtXXT5* (Fig. 7B).

**Fig. 8.**
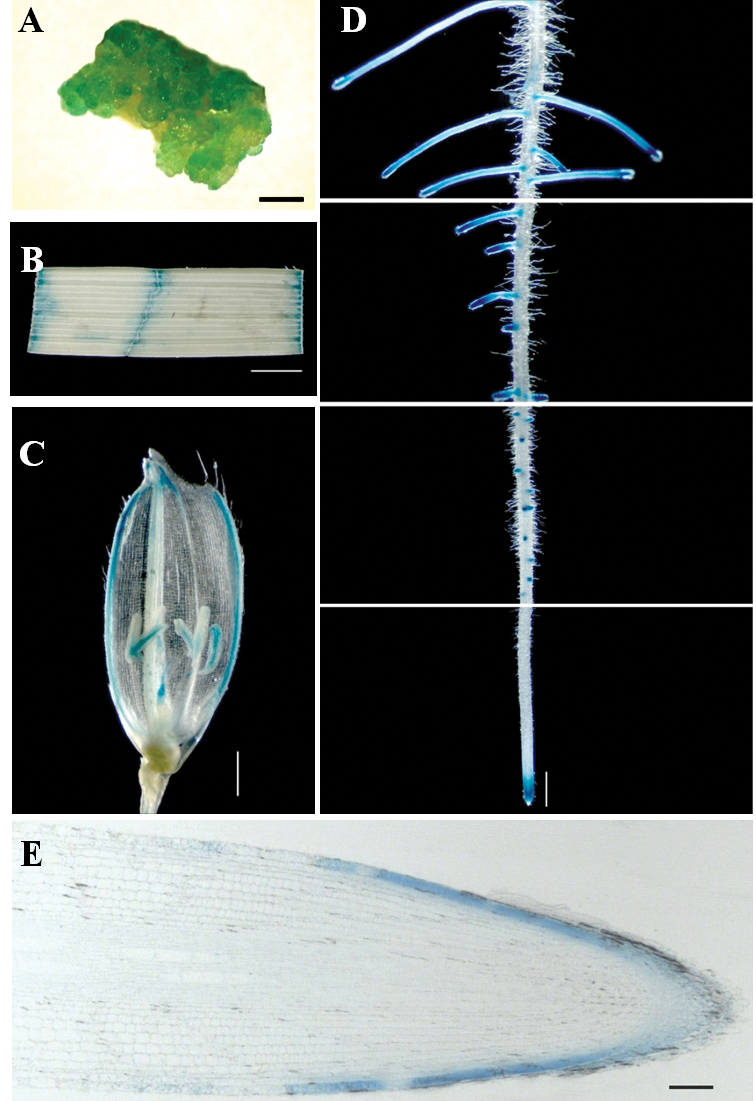
GUS reporter gene expression in transgenic plants expressing the *OsXXT1* promoter-GUS constructs. (A) Callus; (B) Leaf (2-week-old seedlings); (C) Flower (3-month-old seedlings); (D) Root (2-week-old seedlings); (E) Longitudinal section of root tip (2-week-old seedlings). (A–D) Bar=1mm; (E) Bar=20 μm.

To further analyse the expression of *OsXXT1*, a 1.8-kb fragment upstream of the *OsXXT1*start codon was amplified and fused to a GUS reporter gene to make the *OsXXT1*::GUS construct. Using *Agrobacterium*-mediated rice transformation, six independent transgenic lines were generated. Analysis of the T_1_ plants of these transgenic lines showed a similar GUS expression patterns. Expression of GUS was observed in most tissues, including callus, leaves, panicles, inflorescences, and root tips (Fig. 8A–D). Strong GUS staining was observed in the initiating lateral roots (Fig. 8D). Upon development of the lateral roots, the GUS staining was progressively restricted to the tip area (Fig. 8D). Similar expression patterns could be observed in adventitious roots. No GUS staining was detected in the mature zones or root hairs. Horizontal sections of roots showed that the GUS expression was mainly in epidermal cells (Fig. 8D and Fig. S5).

## Discussion

Short root hair phenotypes caused by decreasing XyG content in *Arabidopsis* have been previously reported (Cavalier *et al.*, 2008; Pena *et al.*, 2012; Zabotina *et al.*, 2008; Zabotina *et al.*, 2012). Additionally, it has been previously observed that mutations in the *XXT* gene causes a reduction or elimination of XyG content in *Arabidopsis* cell walls, and could result in a reduced resistance of protoplast pressure and tensile strength of cell walls, and thus lead to a defect in root hair development (Cavalier *et al.*, 2008; Park and Cosgrove, 2012a; Zabotina *et al.*, 2008). In comparison to the XyG content of 20–25% in type I cell walls, cell walls of grass species contain less than 5% XyG. Thus, XyG is considered to be a less important component in type II cell walls (Vogel, 2008). However, our study showed that the mutation of rice *OsXXT1* resulted in a short root hair phenotype, which is similar to the *Arabidopsis xxt1 xxt2* double mutants (Fig. 1). The bubble-like extrusion of root hairs and irregular epidermis cells observed in *srh2* roots under an acidic condition suggested that the tensile strength of cell walls is reduced in the mutant (Fig. 1). OsXXT1 shares high sequence and structural similarity with AtXXT1 and AtXXT2 (Fig. 4, S2). The mutation resulting from EMS mutagenesis is in a conserved domain of XXT proteins and probably resulted in the loss of xyloglucan 6-xylosyltransferase activity in *srh2,* confirmed by the observations that *OsXXT1* gene can complement an *Arabidopsis xxt1 xxt2* double mutant in both root hair growth and XyG biosynthesis. This demonstrates the importance of XyG in type II cell walls.

Although cell walls of grasses only contain a very low amount of XyG, genes encoding XXT and xyloglucan endotransglucosylase/hydrolase (XET/XTH) in rice, barley, and maize genomes are similar to those in *Arabidopsis* (Penning *et al.*, 2009; Strohmeier *et al.*, 2004; Yokoyama *et al.*, 2004). It has been shown that these XET/XTH genes displayed a similar XET/XTH activity in rice as in *Arabidopsis* (Hara *et al.*, 2014). *AtXXT1*, *AtXXT2*, and *AtXXT5* are the major *XXT* genes responsible for XyG biosynthesis (Vuttipongchaikij *et al.*, 2012; Zabotina *et al.*, 2012), which is in accordance with the high expression level of these three genes in most tissues (Fig 7B). Interestingly, the rice *OsXXT1* and *OsGT2* were homologues of *AtXXT1/AtXXT2* and *AtXXT5*, respectively, and displayed similar expression patterns with the *Arabidopsis* genes (Fig 7A, B). Therefore, *OsXXT1* may have a similar function as *AtXXT1*/*AtXXT2* and XyG has an important structural function in both type I and type II cell walls, even though the XyG content is different.

In *Arabidopsis*, the *AtXXT1* and *AtXXT2* display functional redundancy and phylogenic analysis indicate they form a distinct cluster (Vuttipongchaikij *et al.*, 2012). Interestingly, *OsXXT1* is the only gene in rice that branches with this cluster (Fig. 4). This probably explains why a single mutation of *OsXXT1* exhibited a similar short root hair phenotype as the *Arabidopsis xxt1 xxt2* double mutant. Moreover, constitutive expression of *OsXXT1* in the *Arabidopsis xxt1 xxt2* double mutant partially complemented the growth phenotype and XyG synthesis, which demonstrates that OsXXT1 possess xyloglucan 6-xylosyltransferase activity. However, the *Arabidopsis xxt1 xxt2* double mutant complemented with a *35S::OsXXT1* transgenic line contained a relatively low abundance of XyG oligosaccharides, especially lacking galactose modified XyG (XLG and XXLG). This result suggests that the xyloglucan 6-xylosyltransferase activity of *OsXXT1* may not be exactly the same as *AtXXT1* and *AtXXT2*.

Although the proportions and structure of XyG vary between type I and type II cell walls, all flowering plants studied to date contain XyG in their primary cell walls (Hsieh and Harris, 2009). It is has been observed that the XET activity of epidermal cells in root elongation zones and trichoblasts of diverse species of vascular plants is high (Vissenberg *et al.*, 2003). It is possible that the active form of XET is involved in the restructuring of XyG to regulate root hair development in all vascular plants. *OsXXT1* is preferentially expressed in epidermal cells of primary, adventurous, and lateral roots (Fig. 7E, S3). The expression pattern supports the hypothesis that XyG synthesis and modification in root epidermal cells is critical for cell wall development in the root hairs of all plants. Recently, a root hair specific galacturonic acid containing XyG has been discovered in *Arabidopsis* and the lack of this galacturonic acid containing XyG resulted in a short hair phenotype (Pena *et al.*, 2012). Therefore, XyG is a pivotal component in the regulation of root hair tip growth in both *Arabidopsis* and rice.

XyG is the most abundant hemicellulose in type I primary cell walls. This polysaccharide has been depicted as a binding surface of cellulose microfibrils, forming a load-bearing network (Thompson, 2005). However, the important role of XyG in this model has been challenged by the discovery that a lack of XyG in *Arabidopsis* manifests as a short root hair phenotype and an ability to grow normally, despite the defect in root hair morphology (Cavalier *et al.*, 2008). Based on the biomechanical properties of the *Arabidopsis xxt1 xxt2* double mutant and wild-type *Arabidopsis*, a revised architecture of primary cell wall was proposed: a minor XyG component was found in wall mechanics (Park and Cosgrove, 2012b). The analysis of the microarray data and GUS expression patterns under the *OsXXT1* promoter suggest that *OsXXT1* is expressed in a variety of tissues, yet like in *Arabidopsis* (Cavalier *et al.*, 2008; Vuttipongchaikij *et al.*, 2012), a phenotypic defect is only observed in root hair development. If only a minor, inaccessible XyG component works as a load-bearing connection between microfibrils in type I cell walls, it is reasonable to suggest that a relatively low abundance of XyG has a similar function in type II cell walls. Thus, it seems that XyG plays a similar role in the cell walls of both plants with type I and type II cell walls, and it seems that under normal situations, this role is only observed to be non-redundant in root hair development.

## Supplementary data

Supplementary data are available at *JXB* online

Figure S1. Growth performance of *srh2* mutant and wild-type (cv Kasalath, *kas*) plants. The seedlings were growth at nutrient solution (pH 5.5) for 7 d and examined under an electron microscope.

Figure S2. Confirmation the single nucleotide mutation of *srh2* by dCAPS marker. A, The PCR fragment of WT contained three *Nco* I site, whereas mutation of *srh2* eliminate one *Nco*I site (red colour). B, Electrophoresis of *Nco*I-digested PCR product. The red arrows indicated specific digested fragment of wild-type and mutant samples.

Figure S3. Protein sequence alignment of putative xyloglucan 6-xylosyltransferase in *Arabidopsis* and rice. The transmembrane and glycosyltransferase domains were indicated by red box and black line, respectively.

Figure S4. The expression of *OsXXT1* in complementation *Arabidopsis* by RT-PCR.

Figure S5. A, Transverse section of root mature region of OsXXT1::promoter GUS plants. Bar=20 μm.

Table S1. Primers used in this research.

Supplementary Data

## Abbreviations:

dCAPS: derived cleaved amplified polymorphic sequence
GAX: glucuronoarabinoxylans
GT34: glycosyltransferase family 34
GUS: β-glucuronidase
MALDI-TOF: matrix-assisted laser desorption/ionization time-of-flight
MLG: mixed linkage glucans
MS: Murashige and Skoog
srh2: short root hair 2
SSR: simple sequence repeat
Ubi-1: ubiquitin-1
XET/XTH: xyloglucan endotransglucosylase/hydrolase
XXT1: xyloglucan xylosyltransferase1
XyG: xyloglucan.
