# Supplementary material for: Mutation in xyloglucan 6-xylosytransferase results in abnormal root hair development in Oryza sativa
Source: J Exp Bot. 2014 May 15;65(15):4149–57. doi: 10.1093/jxb/eru189 (PMC4112626; doi:10.1093/jxb/eru189)
Supplement: Supplementary Data [file supp_65_15_4149__index.html]

Mutation in xyloglucan 6-xylosytransferase results in abnormal root hair development in Oryza sativa — Mutation in xyloglucan 6-xylosytransferase results in abnormal root hair development in Oryza sativa — Supplementary Data 

# Mutation in xyloglucan 6-xylosytransferase results in abnormal root hair development in *Oryza sativa*

## Supplementary Data

Data files

**Files in this Data Supplement:**

- Supplementary Data - Supplementary Data
