# Supplementary material for: Mutation in xyloglucan 6-xylosytransferase results in abnormal root hair development in Oryza sativa
Source: J Exp Bot. 2014 May 15;65(15):4149–57. doi: 10.1093/jxb/eru189 (PMC4112626; doi:10.1093/jxb/eru189)
Supplement: Supplementary Data [file supp_eru189_jexbot118448_file001.pdf]

Mutation in xyloglucan 6-xylosytransferase results in abnormal root hair development in *Oryza sativa. Chuang Wang, Shuai Li, Sophia Ng, Baocai Zhang, Yihua Zhou, James Whelan, Ping Wu, and Huixia Shou*

SUPPLEMENTARY DATA

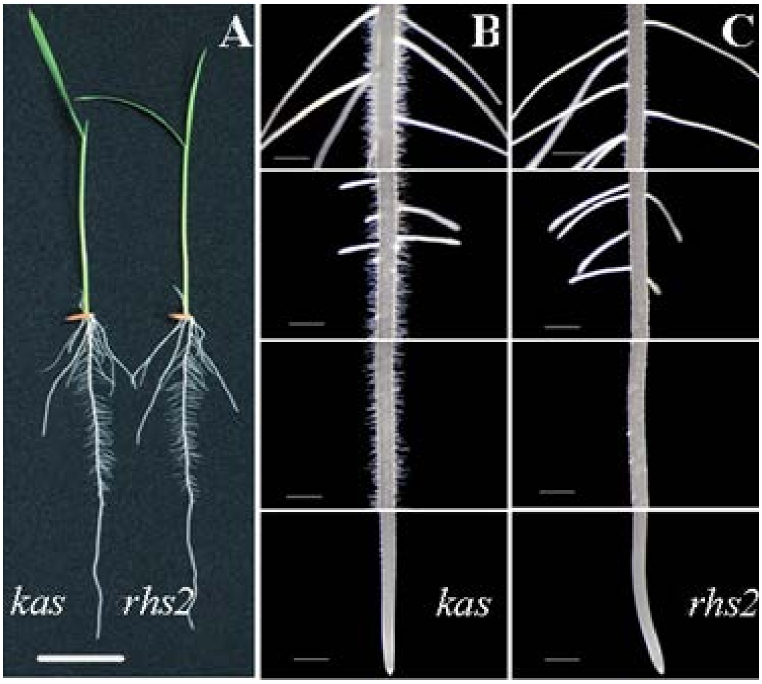

Supplementary Figure S1. Growth performance of *srh2* mutant and wild type (cv Kasalath, *kas*) plants. The seedlings were growth at nutrient solution (pH 5.5) for seven days and examined under an electron microscope examination.

Figure S2

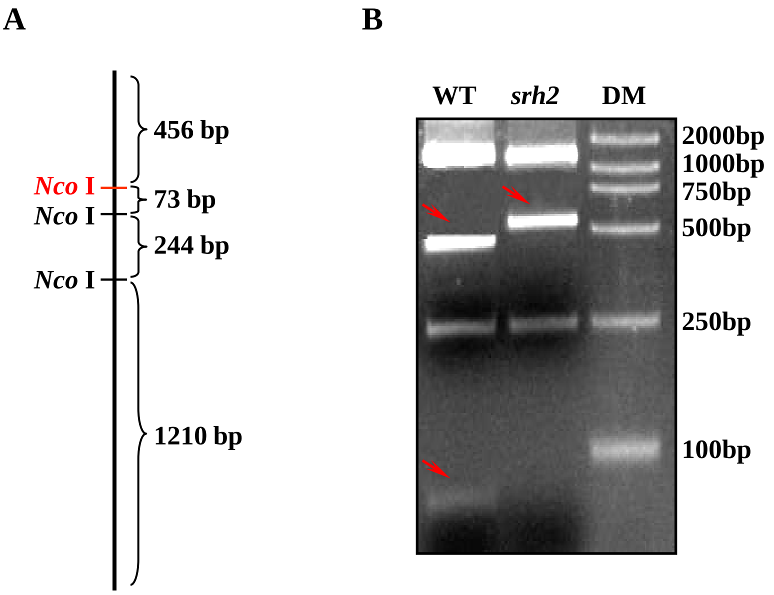

Supplementary Figure S2. Confirmation the single nucleotide mutation of *srh2* by dCAPS marker. A, The PCR fragment of WT contained three *Nco* I site while mutation of *srh2* eliminate one *Nco* I site (red colour). B, Electrophoresis of *Nco* I digeted PCR product. The red arrows indicated specific digested fragment of wild type and mutant samples.

Figure S3

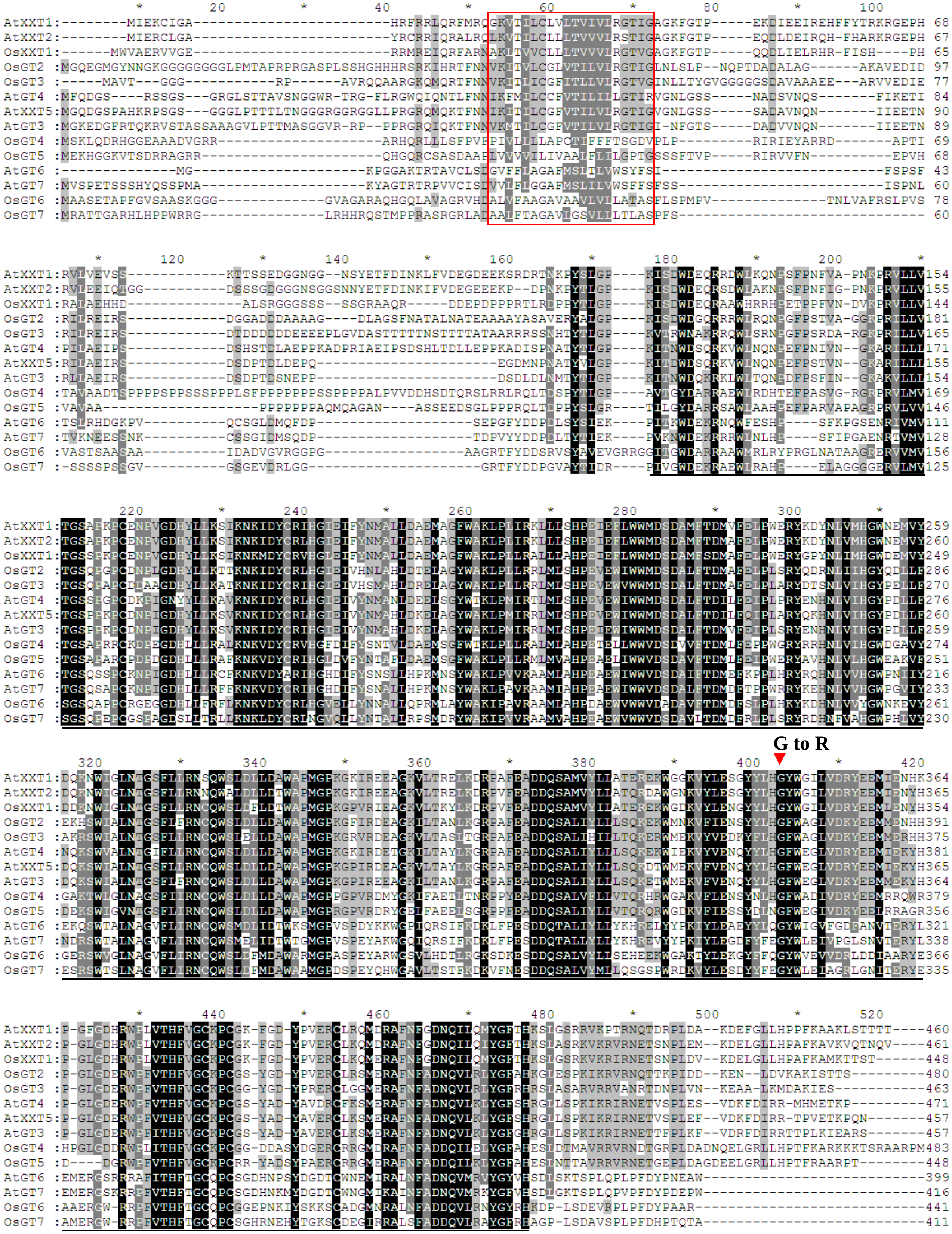

Supplementary Figure S3. Protein sequence alignment of putative xyloglucan 6-xylosyltransferase in Arabidopsis and rice. The transmembrane and glycosyltransferase domains were indicated by red box and black line, respectively.

Figure S4

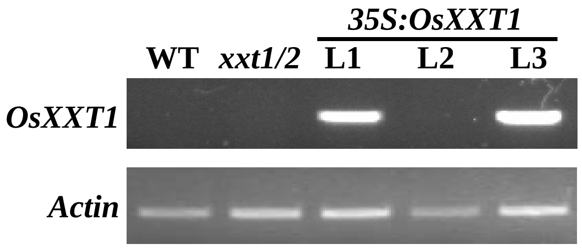

Supplementary Figure S4. The expression of *OsXXT1* in complementation Arabidopsis by RT-PCR.

Figure S5

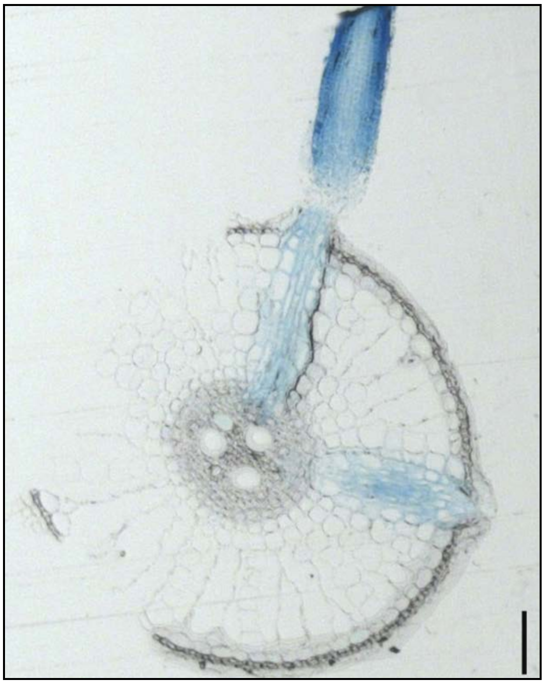

Supplementary Figure S5. A, Transverse section of root mature region of OsXXT1::promoter GUS plants. Bar = 20 μm.

Supplementary Table S1 Primers used in this research

Primer name	Forward (5' to 3')	Reverse (5' to 3')
OsXXT1-Ox	ACCCAAACTCCCTTGTCAACC	CGCGTTGGCTGGCTATTC
OsXXT1-Promoter	ATCGAAGCTTCCATGAAGAAGACCAGTGCTGA	CGATGGATCCCCTCTCCTGCCCCTTCCTC
STS274-03-04	CATCCACATTGTCTGCGGTC	CTCGCTGAAACTCTTGACTG
STS274-04	TGTGGTTAGCCAGCTCCTGG	AACACTCCAGCCAGCGAGCG
STS274-04-06	GATGCAATCTCACCTCAATAC	GGTGATGCAAAGACCTGGTAA
STS274-08	CGCTGTATACTGCATAGCAGTAG	TGAGGTAAGTCATTAGCTCATG
